# Headache Frequency and Depressive Symptoms Are Independently Associated With Disability in Migraine: A Large‐Scale, Cross‐Sectional Study

**DOI:** 10.1155/prm/9964936

**Published:** 2026-08-26

**Authors:** Yunling Pu, Danni Deng, Yuanrong Yao, Feng Hong, Yan Zheng, Mei Mao

**Affiliations:** ^1^ School of Public Health and Health Sciences, Guizhou Medical University, Key Laboratory of Environmental Pollution and Disease Monitoring, Ministry of Education, Guiyang 561113, China, meb.gov.tr; ^2^ Department of Rehabilitation Medicine, Guizhou Provincial People’s Hospital, Guiyang 550002, China, 5055.cn; ^3^ Department of Neurology, Guizhou Provincial People’s Hospital, Guiyang 550002, China, 5055.cn

**Keywords:** comorbidity, depressive symptoms, disability, disease burden, headache frequency, migraine

## Abstract

**Objective:**

The burden of migraine is influenced by a complex interplay of headache characteristics, psychiatric comorbidities, and sleep disturbances. This study aimed to examine factors independently associated with migraine‐related disability and severe functional impairment in a large clinical cohort.

**Methods:**

This cross‐sectional study included 1186 patients diagnosed with migraine according to ICHD‐3 criteria. We collected data on demographics, headache characteristics (monthly headache days [MHD] and intensity [VAS]), and potential medication overuse (pMOH). Disease burden was assessed using the Migraine Disability Assessment (MIDAS) score. Psychiatric comorbidities and sleep quality were evaluated using the Patient Health Questionnaire‐9 (PHQ‐9), Generalized Anxiety Disorder‐7 (GAD‐7), and Pittsburgh Sleep Quality Index (PSQI). Multiple linear and binary logistic regression analyses were performed. Candidate variables were selected a priori based on clinical relevance and data availability. Multicollinearity diagnostics, sensitivity analyses using available medication‐related variables, and model performance assessments were also conducted.

**Results:**

The cohort had a mean age of 39.5 ± 12.1 years, with a high prevalence of moderate‐to‐severe disability (64.7%), anxiety symptoms (52.4%), and poor sleep (48.2%). In the multiple linear regression model (adjusted *R* ^2^ = 0.518, *p* < 0.001), MHD (*β* = 0.581, *p* < 0.001) and PHQ‐9 score (*β* = 0.152, *p* < 0.001) showed the strongest independent associations with the MIDAS score. Headache intensity (VAS) was also significant but had a smaller effect size (*β* = 0.055, *p* = 0.031). GAD‐7 and PSQI were not independently associated with MIDAS after full adjustment. Sensitivity analyses additionally adjusting for available medication‐related variables yielded similar results. The logistic regression model showed acceptable discrimination (AUC = 0.86, 95% CI: 0.83–0.89) and calibration (Hosmer–Lemeshow *χ* ^2^ = 7.42, *p* = 0.492).

**Conclusion:**

In this cross‐sectional cohort of patients with migraine, headache frequency and depressive symptoms were the factors most robustly associated with migraine‐related disability among the available variables. The associations of anxiety symptoms and poor sleep with disability were attenuated after adjustment for headache frequency and depressive symptoms, which may reflect shared variance rather than a confirmed mediation pathway. These findings support routine assessment of headache frequency and depressive symptoms in migraine care, while longitudinal studies with detailed medication and psychosocial data are needed to clarify temporal and causal relationships.

## 1. Introduction

Migraine is a highly prevalent and debilitating neurological disorder, affecting over a billion individuals worldwide and representing a leading cause of disability, particularly among young adults and women during their most productive years [1, 2]. The burden of migraine extends far beyond the physical sensation of pain; it imposes a substantial socioeconomic toll and profoundly impairs patients’ quality of life, affecting their work, family, and social functioning [3–5]. The clinical presentation and impact of migraine are highly heterogeneous, varying in frequency, intensity, and associated symptoms, which contributes to a wide spectrum of disease‐related disability [6–8].

The burden of migraine is frequently compounded by a high prevalence of psychiatric and sleep‐related comorbidities [9–12]. Depressive and anxiety disorders are significantly more common in individuals with migraine than in the general population, with evidence suggesting a complex, bidirectional relationship where each condition can precipitate or exacerbate the other [13, 14]. Similarly, sleep disturbances are a hallmark complaint among migraine patients, acting as both a common trigger for attacks and a consequence of the disorder, thus creating a potential vicious cycle that perpetuates headache and disability [15–17]. The presence of these comorbidities is consistently linked to poorer clinical outcomes, including an increased risk of migraine chronification, greater functional impairment, and reduced treatment response [18, 19].

Furthermore, specific clinical phenotypes, such as chronic migraine (CM) and medication‐overuse headache (MOH), represent a subpopulation of patients who experience a disproportionately high disease burden [19, 20]. The transition from episodic to CM often involves a complex interplay of clinical, psychological, and behavioral factors, yet the relative contribution of these factors to severe disability is not fully understood [21–23]. While the associations between migraine, its comorbidities, and disability are well‐established, the relative importance and independent contributions of these multifaceted factors to the overall disease burden are less well elucidated. It remains unclear whether disability is more strongly associated with core headache characteristics (e.g., frequency and intensity) or with comorbid conditions (e.g., depression, anxiety, and poor sleep), even after accounting for headache severity [24–26]. Clarifying these relationships is important for identifying high‐risk patients and informing more comprehensive clinical assessment strategies.

Therefore, the present study was designed to address this gap by using a large clinical cohort of migraine patients. Our primary objectives were (1) to comprehensively describe the demographic profile, clinical characteristics, and the overall burden of disease, psychiatric comorbidities, and sleep disturbances; (2) to compare these features between key clinical subgroups, including episodic versus CM and patients with versus without potential medication overuse; and (3) most importantly, to employ multivariate regression models to examine factors independently associated with migraine‐related disability and severe functional impairment.

## 2. Methods

### 2.1. Study Design and Participants

This cross‐sectional, observational study was conducted at the Headache Clinic of Guizhou Provincial People’s Hospital between March 2023 and October 2025. Consecutive patients visiting the clinic who were diagnosed with migraine were screened for eligibility. The study was approved by the Institutional Review Board and Ethics Committee of Guizhou Provincial People’s Hospital (approval no: 2023‐017). All participants provided written informed consent before enrollment.

### 2.2. Eligibility Criteria

Participants were included if they met the following criteria:

1. Aged 18 years or older.
2. A confirmed diagnosis of migraine, with or without aura (MWA or MWoA), according to the International Classification of Headache Disorders, 3rd edition (ICHD‐3).
3. A migraine history of at least 12 months.
4. Ability to comprehend and independently complete the study questionnaires.

Exclusion criteria were as follows:

1. Presence of other primary headache disorders (e.g., tension‐type headache or cluster headache) occurring more frequently than migraine.
2. Headache attributed to another disorder (i.e., secondary headache), such as head trauma, vascular disorders, or infection.
3. A diagnosis of a severe, unstable psychiatric disorder (e.g., schizophrenia, bipolar disorder) that could interfere with the assessment.
4. Inability to provide informed consent or complete the questionnaires due to cognitive impairment or language barriers.

### 2.3. Data Collection and Clinical Assessments

Data were collected through a combination of face‐to‐face interviews conducted by trained neurologists and self‐administered questionnaires.

#### 2.3.1. Demographic and Headache Characteristics

Demographic information, including age, sex, and education level, was recorded. Core clinical characteristics of migraine were systematically collected, including disease duration (years), average monthly headache days (MHD) over the past three months, headache intensity during attacks, and migraine subtype (EM or CM; MWA or MWoA).

**Headache intensity** was measured using a 10‐point Visual Analog Scale (VAS), where 0 represented “no pain” and 10 represented “the worst pain imaginable.”

**Episodic migraine (EM)** was defined as having < 15 headache days per month, while **Chronic Migraine (CM)** was defined as having ≥ 15 headache days per month for more than three months, with at least 8 days meeting the criteria for migraine.

#### 2.3.2. Disease Burden Assessment

Migraine‐related disability was assessed using the Migraine Disability Assessment (MIDAS) questionnaire. The MIDAS score quantifies headache‐related disability over the preceding three months across work/school, household, and social domains. Total scores were used to classify disability into four grades: Grade I (0–5, little or no disability), Grade II (6–10, mild disability), Grade III (11‐20, moderate disability), and Grade IV (≥ 21, severe disability).

#### 2.3.3. Psychiatric and Sleep Assessments

**Depressive symptoms** were evaluated using the **Patient Health Questionnaire-9 (PHQ-9)**. It is a 9‐item self‐report scale where each item is scored from 0 (“not at all”) to 3 (“nearly every day”), yielding a total score from 0 to 27.

**Anxiety symptoms** were assessed with the **Generalized Anxiety Disorder-7 (GAD-7)** scale. This 7‐item tool screens for anxiety, with each item scored from 0 to 3, resulting in a total score ranging from 0 to 21. Scores of 5, 10, and 15 represent cut‐off points for mild, moderate, and severe anxiety, respectively.

**Sleep quality** was evaluated using the **Pittsburgh Sleep Quality Index (PSQI)**. The PSQI is a 19‐item self‐rated questionnaire that assesses sleep quality and disturbances over a one‐month period. It yields a global score ranging from 0 to 21, with higher scores indicating poorer sleep quality. For the subgroup analysis, we also used the first component item of the PSQI, which asks patients to rate their overall sleep quality on a 4‐point scale from “very good” to “very bad.”

#### 2.3.4. Assessment of Potential Medication Overuse

Potential medication overuse (pMOH) was identified using a screening question based on the ICHD‐3 criteria for MOH. Patients were classified into the pMOH group if they reported regular intake of simple analgesics on ≥ 15 days/month or combination analgesics, triptans, opioids, or ergots on ≥ 10 days/month for more than three months. All other patients were classified into the non‐MOH group.

### 2.4. Statistical Analysis

All statistical analyses were performed using SPSS (IBM Corp., Armonk, NY, USA). A two‐sided *p* value < 0.05 was considered statistically significant. Continuous variables were tested for normality using the Shapiro–Wilk test. Normally distributed data were presented as mean ± standard deviation (SD), while non‐normally distributed (skewed) data were presented as median and interquartile range (IQR). Categorical variables were described as frequencies and percentages (*n*, %). Differences between groups (EM vs. CM; pMOH vs. non‐MOH) were compared using the independent samples *t*‐test for normally distributed continuous variables or the Mann–Whitney *U* test for non‐normally distributed variables. Differences in disease burden (MIDAS scores) across the four subjective sleep quality groups were assessed using a one‐way analysis of variance (ANOVA), followed by post hoc tests for pairwise comparisons.

To identify factors independently associated with migraine disease burden, a multiple linear regression analysis was performed with the total MIDAS score as the dependent variable. Candidate variables were selected a priori based on clinical relevance, previous literature, and data availability, rather than by automated stepwise selection. These variables included demographic factors, headache‐related characteristics, psychiatric symptoms, sleep disturbance, and migraine subtype. Specifically, MHD, PHQ‐9 score, GAD‐7 score, PSQI score, VAS score, age, sex, disease duration, and migraine subtype were entered into the model. To assess potential multicollinearity among predictors, variance inflation factors (VIFs) and tolerance values were calculated. A VIF value below 5 was considered to indicate the absence of severe multicollinearity. As a sensitivity analysis, we additionally adjusted for available medication‐related variables, including potential medication overuse, previous preventive medication use, and hypnotic medication use assessed by the PSQI medication‐use item. Detailed results of the sensitivity analysis are provided in Supporting Table S1. Detailed information on specific antidepressants, anxiolytics, and individual migraine preventive regimens was not systematically available and was therefore addressed as an important limitation. Finally, a binary logistic regression analysis was conducted to explore factors associated with severe functional disability. The dependent variable was dichotomized as MIDAS Grade IV (severe disability) versus Grades I–III (nonsevere disability). Odds ratios (ORs) with their 95% confidence intervals (CIs) were calculated. For the binary logistic regression model, discriminative ability was evaluated using the area under the receiver operating characteristic curve (AUC), and calibration was assessed using the Hosmer–Lemeshow goodness‐of‐fit test. A nonsignificant Hosmer–Lemeshow test was considered to indicate acceptable model calibration.

## 3. Results

### 3.1. Demographic and Clinical Characteristics of the Study Population

A total of 1186 patients with migraine who met the standard criteria were finally included in this study. The demographic and core clinical characteristics of the study participants are detailed in Table 1. The mean age of the participants was 39.5 ± 12.1 years, with a predominance of females (*n* = 894, 75.4%). The median disease duration was 7.0 years. Regarding headache characteristics, the mean number of MHD was 11.8 ± 8.5 days, and the mean pain intensity during attacks, as measured by the VAS, was 6.7 ± 1.6.

**TABLE 1:** Demographic and clinical characteristics of patients with migraine (*N* = 1186).

Characteristic	Category	Value
*Demographic characteristics*		
Age (years)	—	39.5 ± 12.1

Sex	Male	292 (24.6%)
	Female	894 (75.4%)

Education level	High school or below	458 (38.6%)
	College/bachelor’s degree	627 (52.9%)
	Postgraduate or above	101 (8.5%)

*Core headache clinical characteristics*		
Disease duration (years)	—	7.0 (3.0, 14.0)^1^

Monthly headache days (MHD)	—	11.8 ± 8.5

Headache type	Episodic migraine (EM, < 15 days/month)	815 (68.7%)
	Chronic migraine (CM, ≥ 15 days/month)	371 (31.3%)

Headache intensity (VAS score)	—	6.7 ± 1.6

Migraine subtype	Migraine without aura (MWoA)	1025 (86.4%)
	Migraine with aura (MWA)	161 (13.6%)

*Note:* For continuous variables, normally distributed data are presented as mean ± standard deviation (mean ± SD). Categorical variables are presented as frequency (percentage) [*n* (%)].

^1^Skewed data are presented as median (interquartile range) [M (P25, P75)].

### 3.2. Overall Status of Disease Burden, Psychiatric Comorbidities, and Sleep Disorders

As shown in Table 2, the burdens of disease, anxiety symptoms, and sleep problems were prevalent in our study cohort. Among the 1089 patients with valid MIDAS scores, 64.7% experienced moderate to severe disability (MIDAS ≥ 11). Regarding anxiety, more than half of the patients (52.4%) reported symptoms of varying severity based on the GAD‐7 scale. Subjective sleep quality was also poor, with 48.2% of patients rating their sleep as “fairly bad” or “very bad” on the PSQI self‐rating item.

**TABLE 2:** Distribution of disease burden, anxiety, and subjective sleep quality in patients with migraine.

Variable and classification	Frequency (*n*)	Percentage (%)
*Migraine disability assessment (MIDAS) (N = 1089)*		
Grade I (little or no disability)	384	35.3
Grade II (mild disability)	179	16.4
Grade III (moderate disability)	207	19.0
Grade IV (severe disability)	319	29.3

*Anxiety symptom severity (GAD-7) (N = 1172)*		
None/minimal anxiety (0–4)	658	56.1
Mild anxiety (5–9)	375	32.0
Moderate anxiety (10–14)	71	6.1
Severe anxiety (≥ 15)	68	5.8

*Subjective sleep quality (PSQI item) (N = 1165)*		
Very good	156	13.4
Fairly good	448	38.5
Fairly bad	460	39.5
Very bad	101	8.7

*Note:* The valid sample size (*N*) for each variable differs due to missing data. Percentages are calculated based on the valid sample size.

### 3.3. Subgroup Analysis and Univariate Comparisons

#### 3.3.1. Comparison of Characteristics Between Patients With Chronic and Episodic Migraine

Table 3 presents the differences between the EM and CM groups. Compared to patients with EM, patients with CM had significantly higher scores for disease burden (MIDAS), depression (PHQ‐9), anxiety (GAD‐7), and sleep disturbance (PSQI), as well as higher headache intensity (VAS) (all *p* < 0.001).

**TABLE 3:** Comparison of clinical and psychological characteristics between patients with episodic (EM) and chronic (CM) migraine.

Variable	EM group (*n* = 815)	CM group (*n* = 371)	*t* value	*p* value
	Mean ± SD	Mean ± SD		
Monthly headache days	6.2 ± 3.0	24.1 ± 4.2	−62.55	< 0.001
MIDAS total score	18.9 ± 10.1	47.1 ± 11.5	−38.12	< 0.001
PHQ‐9 total score	7.8 ± 5.1	12.9 ± 5.5	−15.99	< 0.001
GAD‐7 total score	7.2 ± 5.0	11.2 ± 5.1	−12.43	< 0.001
PSQI total score	8.8 ± 4.0	12.1 ± 4.1	−12.58	< 0.001
VAS score	6.4 ± 1.5	7.3 ± 1.6	−9.11	< 0.001

*Note:* Independent samples *t*‐test was used. Z value was used for variables with skewed distributions where appropriate.

#### 3.3.2. Disease Burden Stratified by Subjective Sleep Quality

**FIGURE 1:**
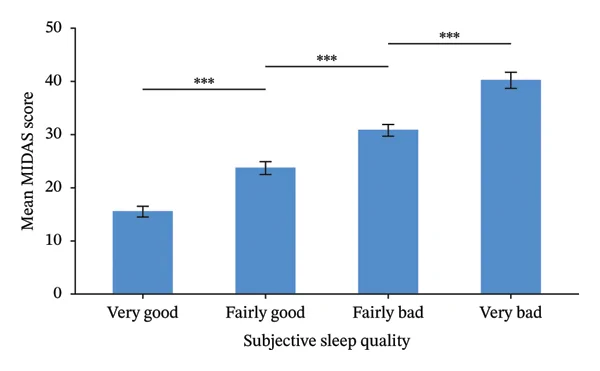
Migraine disease burden stratified by subjective sleep quality. The bar chart displays the mean migraine disability assessment (MIDAS) scores across four categories of self‐reported sleep quality. A clear stepwise increase in migraine‐related disability is observed as sleep quality worsens. Error bars represent the standard error of the mean (SEM). A one‐way ANOVA showed a significant overall effect (*F* = 198.7, *p* < 0.001). Post hoc tests confirmed significant differences between each adjacent group.^∗∗∗^ *p* < 0.001.

Figure 1 visually demonstrates the change in migraine disease burden (MIDAS score) with varying levels of subjective sleep quality. A one‐way ANOVA revealed that the mean MIDAS score showed a significant, stepwise increase as subjective sleep quality worsened (*F* = 198.7, *p* < 0.001). Patients who rated their sleep as “very good” had a mean MIDAS score of only 15.5, whereas those who rated it as “very bad” had a mean score of 40.2. This significant difference suggests a strong association between sleep quality and the degree of headache‐related disability.

### 3.4. Multivariate Linear Regression Analysis of Factors Influencing Migraine Disease Burden

To quantitatively analyze the independent contribution of various factors to migraine disease burden, a multiple linear regression model was constructed. The final model results are presented in Table 4. The overall model was statistically significant (*F* = 171.3, *p* < 0.001), with an adjusted *R* ^2^ of 0.518, indicating that the independent variables in the model could jointly explain 51.8% of the variance in the MIDAS score. After controlling for other variables, MHD (*β* = 0.581, *p* < 0.001) and depressive symptoms (PHQ‐9 total score) (*β* = 0.152, *p* < 0.001) showed the strongest independent positive associations with the MIDAS score. Headache intensity (VAS) (*β* = 0.055, *p* = 0.031) was also independently associated with the MIDAS score, although its effect size was smaller. GAD‐7 (*p* = 0.102) and PSQI (*p* = 0.188) were not independently associated with MIDAS in the fully adjusted model. This finding should be interpreted cautiously, because it may partly reflect shared variance among depressive symptoms, anxiety symptoms, sleep disturbance, and headache frequency.

**TABLE 4:** Multiple linear regression analysis predicting migraine disease burden (MIDAS total score) (*N* = 1186).

Independent variable	B	SE	*β*	*t*	*p* value	95% confidence interval (CI)
Monthly headache days (MHD)	**1.21**	**0.03**	**0.581**	**34.95**	**< 0.001**	**1.15, 1.27**
PHQ‐9 total score	**0.423**	**0.069**	**0.152**	**6.130**	**< 0.001**	**0.288, 0.558**
Headache intensity (VAS)	**0.556**	**0.257**	**0.055**	**2.163**	**0.031**	**0.051, 1.061**
GAD‐7 total score	0.142	0.087	0.048	1.637	0.102	−0.028, 0.312
PSQI total score	0.145	0.108	0.039	1.316	0.188	−0.067, 0.357
Age (years)	−0.021	0.028	−0.018	−0.750	0.453	−0.076, 0.034
Sex (female vs. male)	0.899	0.701	0.029	1.282	0.200	−0.476, 2.274
Disease duration (years)	0.033	0.035	0.022	0.943	0.346	−0.036, 0.102
Migraine subtype (MWA vs. MWoA)	1.350	0.985	0.031	1.371	0.171	−0.583, 3.283

*Note:* B, unstandardized regression coefficient; *β*, standardized regression coefficient. Model adjusted *R* ^2^ = 0.518. Bold indicates *p* < 0.05.

Abbreviation: SE, standard error.

Multicollinearity diagnostics indicated that all predictors had VIF values below 5. The VIF values were 1.42 for MHD, 2.63 for PHQ‐9, 2.84 for GAD‐7, 1.76 for PSQI, 1.18 for VAS, 1.09 for age, 1.06 for sex, 1.12 for disease duration, and 1.04 for migraine subtype, suggesting that severe multicollinearity was unlikely to account for the main findings. Detailed multicollinearity diagnostics are presented in Supporting Table S2.

As a sensitivity analysis, we additionally adjusted for available medication‐related variables, including potential medication overuse, previous preventive medication use, and hypnotic medication use. MHD and PHQ‐9 score remained independently associated with both the MIDAS total score and severe disability. In the sensitivity linear regression model, MHD showed the largest standardized coefficient (*β* = 0.553, *p* < 0.001), followed by PHQ‐9 score (*β* = 0.146, *p* < 0.001). In the sensitivity logistic regression model, MHD (OR = 1.19, 95% CI: 1.16–1.23, *p* < 0.001) and PHQ‐9 score (OR = 1.15, 95% CI: 1.11–1.19, *p* < 0.001) remained significant factors associated with severe disability.

### 3.5. Visualization of Regression Analysis Results

**FIGURE 2:**
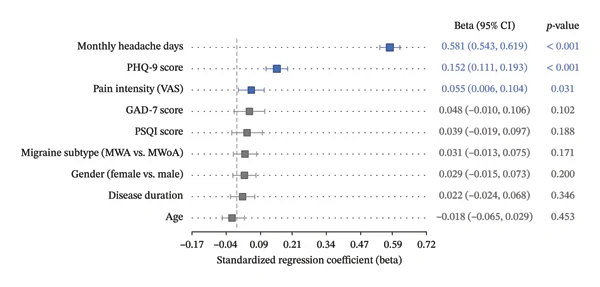
Forest plot of standardized regression coefficients (β) for predictors of migraine disease burden. The plot visualizes the results from the multiple linear regression model predicting the migraine disability assessment (MIDAS) score. Each square represents the standardized β coefficient, indicating the strength and direction of the association. The horizontal lines represent the 95% confidence intervals (CIs). Variables with 95% CIs that do not cross the vertical dashed line at zero are statistically significant independent predictors. Blue squares indicate significant predictors (*p* < 0.05), while gray squares indicate nonsignificant predictors.

To better illustrate the relative influence of each factor on migraine‐related disability, the results of the multiple linear regression model were visualized using a forest plot (Figure 2). The plot displays the standardized regression coefficients (β), which allow for direct comparison of the effect sizes of different predictors. As shown, MHD had the largest association with the MIDAS score (*β* = 0.581, 95% CI: 0.543–0.619). Depressive symptoms (PHQ‐9 score) also demonstrated a significant independent association (*β* = 0.152, 95% CI: 0.111–0.193). Headache intensity (VAS) was statistically significant but had a smaller association (*β* = 0.055, 95% CI: 0.006–0.104). In contrast, the 95% CIs for other variables, including anxiety (GAD‐7), sleep quality (PSQI), and demographic factors, crossed the null value of zero, indicating that they were not independently associated with MIDAS in the fully adjusted model.

### 3.6. Impact of Medication Overuse on Disease Burden and Comorbidities

To investigate the association between potential medication overuse (pMOH) and clinical burden, patients were divided into a pMOH group (*n* = 289) and a non‐MOH group (*n* = 897) based on a screening item for analgesic use. As shown in Table 5, significant differences were observed between the two groups across all core clinical and psychological metrics. Compared to the non‐MOH group, patients in the pMOH group had significantly more MHD (23.5 vs. 8.1 days, *p* < 0.001), a finding consistent with the clinical profile of MOH. The disease burden (MIDAS total score) in the pMOH group was nearly double that of the non‐MOH group (45.9 vs. 22.3, *p* < 0.001). Furthermore, scores for depression (PHQ‐9), anxiety (GAD‐7), and sleep disturbance (PSQI) were all significantly higher in the pMOH group (all *p* < 0.001). These findings suggest that pMOH identifies a subgroup with higher headache frequency, heavier disability burden, and more severe psychiatric and sleep‐related symptoms.

**TABLE 5:** Comparison of characteristics between potential medication overuse (pMOH) and non‐MOH groups.

Variable	Non‐MOH group (*n* = 897)	pMOH group (*n* = 289)	t Value	*p* Value
	Mean ± SD	Mean ± SD		
Monthly Headache Days	8.1 ± 6.2	23.5 ± 5.1	−39.85	< 0.001
MIDAS Total Score	22.3 ± 12.8	45.9 ± 13.1	−26.71	< 0.001
PHQ‐9 Total Score	8.2 ± 5.3	12.7 ± 5.9	−11.98	< 0.001
GAD‐7 Total Score	7.5 ± 5.1	11.0 ± 5.4	−9.82	< 0.001
PSQI Total Score	9.1 ± 4.1	11.9 ± 4.2	−10.15	< 0.001
VAS Score	6.5 ± 1.5	7.2 ± 1.6	−6.88	< 0.001

*Note:* Independent samples *t*‐test was used.

### 3.7. Factors Associated With Severe Functional Disability (MIDAS Grade IV)

**FIGURE 3:**
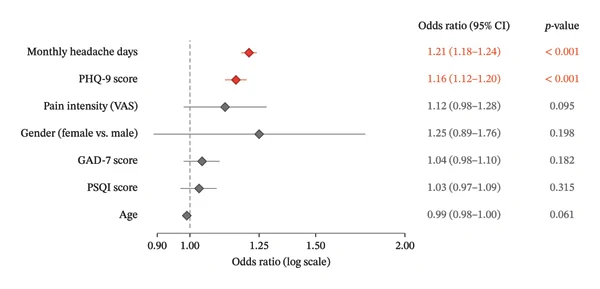
Forest plot of factors associated with severe migraine disability (MIDAS grade IV). This plot illustrates the odds ratios (ORs) and their corresponding 95% confidence intervals (CIs) from the binary logistic regression analysis. The model identifies factors associated with severe migraine‐related disability (MIDAS Grade IV vs. Grades I–III). Each diamond represents the point estimate of the OR, and the horizontal line represents its 95% CI. Variables whose CIs do not cross the vertical line of no effect (OR = 1.0) are statistically significant. Monthly headache days and PHQ‐9 score were the only significant factors in the model and are highlighted in red (*p* < 0.001).

To further identify factors associated with the most severe functional impairment, a binary logistic regression model was constructed, with the presence of severe disability (MIDAS Grade IV vs. Grades I–III) as the dependent variable. The same core variables as in the preceding linear regression were included in the model. The results (Table 6) showed that MHD (OR = 1.21, 95% CI: 1.18–1.24, *p* < 0.001) and PHQ‐9 total score (OR = 1.16, 95% CI: 1.12–1.20, *p* < 0.001) were independently associated with severe disability. Specifically, each additional headache day per month was associated with a 21% increase in the odds of severe disability, and each one‐point increase in the PHQ‐9 score was associated with a 16% increase in the odds of severe disability. Consistent with the linear regression results, anxiety, sleep disturbance, age, and sex were not independently associated with severe disability after controlling for other variables. The forest plot in Figure 3 visually represents the ORs for each factor.

**TABLE 6:** Binary logistic regression analysis of risk factors for severe functional disability (MIDAS grade IV).

Independent variable	OR (odds ratio)	95% CI for OR	*p* value
Monthly Headache Days	**1.21**	**(1.18, 1.24)**	**< 0.001**
PHQ‐9 Total Score	**1.16**	**(1.12, 1.20)**	**< 0.001**
Headache Intensity (VAS)	1.12	(0.98, 1.28)	0.095
GAD‐7 Total Score	1.04	(0.98, 1.10)	0.182
PSQI Total Score	1.03	(0.97, 1.09)	0.315
Age (years)	0.99	(0.98, 1.00)	0.061
Sex (Female vs. Male)	1.25	(0.89, 1.76)	0.198

*Note:* Bold indicates *p* < 0.05.

Abbreviations: CI, confidence interval; OR, odds ratio.

The logistic regression model demonstrated acceptable discriminative ability for severe disability, with an AUC of 0.86 (95% CI: 0.83–0.89). The Hosmer–Lemeshow goodness‐of‐fit test was not significant (*χ* ^2^ = 7.42, *p* = 0.492), suggesting acceptable model calibration. Detailed performance metrics are provided in Supporting Table S3.

## 4. Discussion

In this large, cross‐sectional study of 1186 migraine patients, we comprehensively evaluated the interplay between headache characteristics, psychiatric comorbidities, sleep disturbances, and disease burden. Our findings confirm that migraine imposes a substantial burden, with a high prevalence of disability, depression, anxiety, and poor sleep. Through multivariate modeling, we found that monthly headache frequency and depressive symptoms were the two factors most robustly associated with migraine‐related disability among the available variables. This pattern was observed for both the overall MIDAS score and severe functional impairment, but it should be interpreted as an association rather than evidence of causality.

The central finding of our study is the strong independent association of MHD and PHQ‐9 scores with migraine disability. The standardized coefficient for MHD in our linear regression model was substantially larger than that of other variables, indicating that headache frequency is closely related to functional impairment. This is clinically intuitive and aligns with previous research highlighting headache frequency as a cornerstone of migraine management and a primary outcome in clinical trials [24]. Our logistic regression analysis further showed that each additional headache day per month was associated with higher odds of severe disability. This finding emphasizes the importance of accurately assessing headache frequency in patients with migraine.

Beyond headache frequency, depressive symptoms were also independently associated with migraine disability. Even after controlling for headache frequency, intensity, and other comorbidities, a higher PHQ‐9 score was associated with a higher MIDAS score and a greater likelihood of severe disability. This finding suggests that depressive symptoms may serve as an important clinical correlate of higher disability burden in migraine patients. Potential explanations include shared neurobiological mechanisms, lower pain thresholds, reduced coping ability, and greater perceived impact of headache symptoms. However, because of the cross‐sectional design and the lack of complete medication and psychosocial data, this association should not be interpreted as proving that depression causally drives disability [27, 28].

One noteworthy finding was that anxiety and sleep disturbances (measured by GAD‐7 and PSQI total scores), despite being highly prevalent and showing strong univariate associations with disability, were not independently associated with MIDAS in the fully adjusted regression models. The attenuation of GAD‐7 and PSQI in the fully adjusted model should not be interpreted as evidence that anxiety or sleep disturbance is clinically unimportant, nor as proof of a formal mediation pathway. Rather, this finding may reflect overlapping symptom domains and shared variance among depressive symptoms, anxiety symptoms, sleep disturbance, and headache frequency. Therefore, anxiety and sleep problems remain clinically relevant in migraine management, although their independent statistical associations with MIDAS were weakened after simultaneous adjustment for other closely related factors.

Our subgroup analyses also showed that the burden was higher in specific clinical subgroups. Patients with CM and those with potential medication overuse (pMOH) exhibited higher headache frequency, disability, and psychiatric and sleep scores than their comparison groups. The MIDAS score in the CM group was more than double that of the EM group, which is consistent with the much higher headache frequency observed in the CM group.

The comparison between pMOH and non‐MOH groups was also clinically relevant. The high headache frequency (23.5 days/month) and higher MIDAS score in the pMOH group highlight the heavier burden experienced by patients with potential medication overuse. Because pMOH was identified using a screening item rather than a formal diagnostic interview or prospective headache diary, these findings should be interpreted as indicating an association between possible medication overuse and higher disease burden rather than confirming a causal effect.

Our findings have several important clinical implications. First, they support careful assessment of headache frequency when evaluating migraine‐related disability. Second, screening for depressive symptoms using validated tools such as the PHQ‐9 may help identify patients with higher disability burden. Third, anxiety and sleep problems remain important clinical concerns, even though their independent statistical associations with MIDAS were attenuated in the fully adjusted model. Finally, in patients with high‐frequency headaches, screening for potential medication overuse may help clinicians identify a subgroup with particularly high clinical burden.

This study has several strengths, including its large sample size, the use of validated and standardized assessment tools, and the application of comprehensive multivariate analyses to disentangle complex relationships. However, several limitations must be acknowledged. First, the cross‐sectional design precludes any conclusions about causality. We can identify associations, but longitudinal studies are needed to confirm temporal relationships (e.g., whether depressive symptoms precede disability or whether severe disability contributes to depressive symptoms). Second, our data were collected from a single tertiary headache center, which may have resulted in selection bias toward more severe and complex cases, potentially limiting the generalizability of our findings to the broader migraine population in the community. Third, the diagnosis of pMOH was based on a screening question rather than a formal diagnostic interview and headache diary review, which may have led to some misclassification. Fourth, although we performed a sensitivity analysis using available medication‐related variables, detailed information on specific antidepressants, anxiolytics, and individual migraine preventive regimens was not systematically collected. Therefore, residual confounding related to medication exposure cannot be fully excluded. Fifth, several social and psychological factors, including socioeconomic status, occupational stress, family support, and health‐care access, were not systematically collected in this study. These unmeasured factors may be associated with both migraine attacks and disability and may therefore contribute to residual confounding. Finally, our reliance on self‐report questionnaires is subject to recall and reporting bias.

## 5. Conclusion

In conclusion, in this large cross‐sectional cohort of patients with migraine, MHD and PHQ‐9 score were the factors most robustly associated with migraine‐related disability and severe functional impairment among the available variables. Anxiety symptoms and poor sleep were common and clinically relevant, but their independent associations with disability were attenuated after adjustment for headache frequency, depressive symptoms, and other covariates. These findings support routine assessment of headache frequency and depressive symptoms in migraine care, while future longitudinal studies with detailed medication and psychosocial data are needed to clarify temporal and causal relationships.

## Author Contributions

Yuanrong Yao and Feng Hong proposed the main research objectives. Yunling Pu and Danni Deng were responsible for the study conception and design, study implementation, and manuscript writing. Yunling Pu, Danni Deng, Yan Zheng, and Mei Mao contributed to data collection and organization, statistical analysis, and the preparation of figures and tables. Yunling Pu was responsible for manuscript revision. Yuanrong Yao supervised the study, was responsible for quality control and review, and takes final responsibility for the manuscript.

## Funding

This work was supported by the National Key Research and Development Program of China under Grant No. 2023YFC2508701 (Research on Prevention and Treatment of Common Diseases).

## Disclosure

All authors approved the final version of the manuscript.

## Conflicts of Interest

The authors declare no conflicts of interest.

## Supporting Information

Additional supporting information can be found online in the Supporting Information section.

## Supporting information


**Supporting Information** The following Supporting information is available: Supporting Table S1, sensitivity analysis additionally adjusting for available medication‐related variables; Supporting Table S2, multicollinearity diagnostics for the fully adjusted regression model; and Supporting Table S3, performance of the logistic regression model for severe migraine‐related disability.

## Data Availability

The data that support the findings of this study are available from the corresponding author upon reasonable request.
