# Supplementary material for: Headache Frequency and Depressive Symptoms Are Independently Associated With Disability in Migraine: A Large‐Scale, Cross‐Sectional Study
Source: Pain Res Manag. 2026 Aug 26;2026:9964936. doi: 10.1155/prm/9964936 (PMC13519303; doi:10.1155/prm/9964936)
Supplement: Supplementary file 1 [file PRM-2026-9964936-s001.docx]

**Supplementary Tables**

**Supplementary Table S1. Sensitivity analysis additionally adjusting for available medication-related variables**

| **Model** | **Variable** | **Effect estimate** | **95% CI** | **P value** |
| --- | --- | --- | --- | --- |
| Linear regression, MIDAS total score | Monthly headache days | β = 0.553 | 0.516-0.590 | <0.001 |
| Linear regression, MIDAS total score | PHQ-9 score | β = 0.146 | 0.106-0.187 | <0.001 |
| Linear regression, MIDAS total score | VAS score | β = 0.052 | 0.004-0.101 | 0.039 |
| Linear regression, MIDAS total score | Potential medication overuse | β = 0.071 | 0.021-0.121 | 0.006 |
| Linear regression, MIDAS total score | Previous preventive medication use | β = 0.028 | -0.014-0.069 | 0.188 |
| Linear regression, MIDAS total score | Hypnotic medication use | β = 0.034 | -0.006-0.074 | 0.096 |
| Logistic regression, severe disability | Monthly headache days | OR = 1.19 | 1.16-1.23 | <0.001 |
| Logistic regression, severe disability | PHQ-9 score | OR = 1.15 | 1.11-1.19 | <0.001 |
| Logistic regression, severe disability | Potential medication overuse | OR = 1.42 | 1.06-1.91 | 0.018 |
| Logistic regression, severe disability | Previous preventive medication use | OR = 1.17 | 0.84-1.63 | 0.346 |
| Logistic regression, severe disability | Hypnotic medication use | OR = 1.21 | 0.90-1.64 | 0.214 |

**Supplementary Table S2. Multicollinearity diagnostics for the fully adjusted regression model**

| **Variable** | **VIF** | **Tolerance** |
| --- | --- | --- |
| Monthly headache days | 1.42 | 0.704 |
| PHQ-9 score | 2.63 | 0.380 |
| GAD-7 score | 2.84 | 0.352 |
| PSQI score | 1.76 | 0.568 |
| VAS score | 1.18 | 0.847 |
| Age | 1.09 | 0.917 |
| Sex | 1.06 | 0.943 |
| Disease duration | 1.12 | 0.893 |
| Migraine subtype | 1.04 | 0.962 |

**Supplementary Table S3. Performance of the logistic regression model for severe migraine-related disability**

| **Performance metric** | **Value** |
| --- | --- |
| AUC | 0.86 |
| 95% CI for AUC | 0.83-0.89 |
| Hosmer-Lemeshow χ² | 7.42 |
| Hosmer-Lemeshow P value | 0.492 |
